# Cytokines derived from innate lymphoid cells assist *Helicobacter hepaticus* to aggravate hepatocellular tumorigenesis in viral transgenic mice

**DOI:** 10.1186/s13099-019-0302-0

**Published:** 2019-05-15

**Authors:** Xiao Han, Tianren Huang, Junqing Han

**Affiliations:** 1 Department of Experiment, Tumor Hospital Affiliated to Guangxi Medical University, 71# Hedi Road, Nanning, 530021 China; 2 Department of Tumor Research and Therapy Center, Shandong Provincial Hospital Affiliated to Shandong University, 324# Jingwu Road, Jinan, 250021 China

**Keywords:** *Helicobacter hepaticus*, Hepatitis B virus, Hepatocellular carcinoma, Innate lymphoid cells, IFN-γ/p-STAT1 axis

## Abstract

**Background:**

Recently, intestinal microbiome has been involved in hepatic diseases due to the immunologic and metabolic communication between liver and intestine. Initiation of hepatocellular carcinoma (HCC) frequently attributes to conspiracy between immune cells and infectious carcinogens. Here, the hypothesis that the tumorigenesis of HCC with HBV infection will be aggravated by specific intestinal bacteria was verified in viral transgenic mouse models.

**Methods:**

Comparative 16S rRNA sequencing was adopted to observe the intestinal enrichment of *Helicobacter hepaticus* in HCC. Oral administration of *Helicobacter hepaticus* was carried out to evaluate its hepatic carcinogenic effect in HBV transgenic mice or wildtype C57BL/6. The livers of experimental mice were collected and examined for the degree of tumorigenesis.

**Results:**

We found that *Helicobacter hepaticus* more likely colonized at lower colon of HBV-infected mice with HCC, compared with C57BL/6 and HBV-infected mice without neoplasm. Pretreatment of *Helicobacter hepaticus* in transgenic mice aggravated tumor formation, with higher incidence, more tumor nodule and higher serum AFP. Then, a cytokines expression patterns with inclined IFN-γ, IFN-γR1, IL-17 and IL-23 was found in HBV-infected mice with *Helicobacter hepaticus*. Furthermore, innate lymphoid cells, especially Th17 and NK cells which can secret IL-17 and IFN-γ respectively, might be recruited by *Helicobacter hepaticus* cooperated with HBV. Besides, increased expression of CD69, NKG2D and IFN-γ showed activation of cytokine production in intrahepatic NK cells. Finally, IFN-γ decreased E-cadherin expression through p-STAT1 pathway, resulting in epithelial–mesenchymal transition with inclined expression of Snail2, SIP1 and CXCR4 in vitro. p-STAT1 inhibitor was able to reverse the expression of E-cadherin and EMT resulted from IFN-γ function on HBsAg-positive hepatocytes.

**Conclusions:**

*Helicobacter hepaticus* generate a detrimental immune microenvironment by IFN-γ/p-STAT1 axis which can promote the tumorigenesis of hepatitis B via recruiting innate lymphoid cells.

**Electronic supplementary material:**

The online version of this article (10.1186/s13099-019-0302-0) contains supplementary material, which is available to authorized users.

## Introduction

In accordance with latest statistics, hepatocellular carcinoma (HCC) is the third cause of cancer death in the world [1]. Hepatitis B virus (HBV) infection is associated with at least 55% incidence of cirrhosis, followed by hepatic functional decompensation leading to initiation of HCC [2]. Nowadays, progression of HCC can be significantly restrained by viral polymerase inhibition from nucleotide analogues drugs [3], however, hepatic cancer risk of patients with long-duration HBV infection can remain above normal after functional clearance of the virus infection [4–6]. This clinical problem indicated that some viral integration mechanisms resulting in genomic changes render hepatic carcinogenesis, including chromosomal instability [7, 8], and function regulation of human telomerase reverse transcriptase gene [9]. Moreover, investigators believed that incontrollable inflammation and liver remodeling are highly relative to antiviral immunity [10, 11], considering HBV itself is non-cytopathic to host cells. Therefore, the immune against viral integrated-hepatocytes is vital in the pathogenesis of HBV-associated HCC.

Recently a study demonstrated that intestinal *Lactobacillus gasseri* triggered the production of interleukin 17 by intrahepatic γδ T cells, resulting in cholestatic hepatitis [12]. It seems a few strains with disrupted intestinal barriers can participate in pathogenesis of hepatic disease, including hepatocellular carcinoma, by regulating innate lymphoid cells (ILCs) and their inflammatory chemokines [13–15]. Manipulating intestinal microbiome in mice induced CXCL16 expression of liver sinusoidal endothelial cells, which accumulated CXCR6^+^ natural killer T, reinforcing an antitumor effect by interferon-γ secretion [16]. Evidences for the connection between intestinal microflora and HCC gradually rise, however, few study has investigated the role of specific strains in development of HCC taking viral etiology into consideration. In our study, we used HBV transgenic mice to simulate the course of human HCC, to verify the hypothesis that *Helicobacter hepaticus*, a specific strain highly relative to hepatitis, could facilitate the carcinogenesis of HBV infected hepatitis via regulating innate lymphoid cells.

## Results

### *Helicobacter hepaticus* are over-presented in feces from mice with HBV infected HCC

**Fig. 1:**
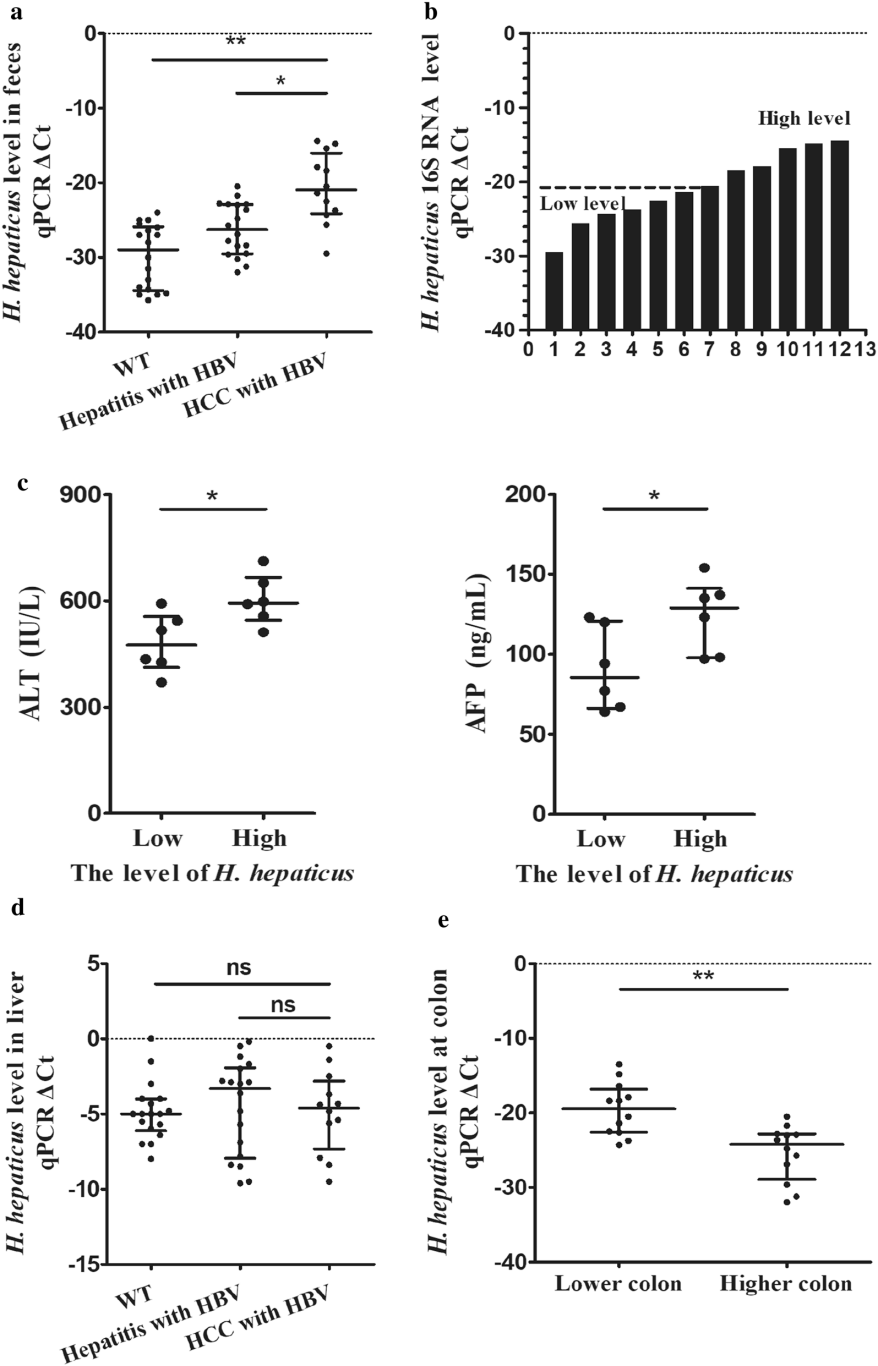
*Helicobacter hepaticus* are over-presented in feces from mice with HBV infected HCC. **a** Abundance of fecal *H. hepaticus* from the control (C57BL/6) (n = 18), HBs-Tg mice without neoplasm (n = 18), and mice with HBV infected HCC (n = 12). **b** Division of *H. hepaticus* abundance into high (n = 6) and low (n = 6) basing on the median value. **c** Comparison of serum ALT, AFP activity between low and high level of fecal *H. hepaticus* in HBs-Tg mice with HCC. **d** Abundance of hepatic *H. hepaticus* from the control (C57BL/6) (n = 18), HBs-Tg mice without neoplasm (n = 18), and mice with HBV infected HCC (n = 12). **e** Comparison of *H. hepaticus* abundance between lower and higher colon in HBs-Tg mice with HCC. Data are expressed as median ± percentile. Mann–Whitney U was used. P < 0.05 represents statistical difference. *P < 0.05, **P < 0.01

Considering the concentrated *Helicobacter* spp. and its carcinogenesis in colorectal cancer [17, 18], we studied whether *Helicobacter hepaticus* in intestine involved in neoplastic development via enterohepatic crosstalk among natural HBs-Tg mice. We analyzed *H. hepaticus* level in feces from the mice at 24–25-month-old with an incidence of 40% for neoplasia as previous studies [19]. *H. hepaticus* was concentrated in feces from mice with hepatic carcinoma compared with that from purely HBV infection (P < 0.01) (Fig. 1a). Based on the median value of *H. hepaticus* level in feces, the cancerous group was divided into high (n = 6) and low (n = 6) abundance group (Fig. 1b). The relation between *H. hepaticus* abundance and the serological hepatic patterns are evaluated. Results showed that higher enrichment of *H. hepaticus* is more likely associated with advanced HCC (ALT P < 0.05, AFP P < 0.05) (Fig. 1c). Moreover, no difference of *H. hepaticus* abundance in the liver was observed between HBV-associated hepatitis and hepatocarcinoma (P > 0.05) (Fig. 1d). S16RNA qPCR analysis to tissues revealed that *H. hepaticus* more likely over-presented at lower colon than higher one (P < 0.01) (Fig. 1e). These data suggest that *H. hepaticus* accumulate at the lower colon of HBV infected HCC, which indicate the *H. hepaticus* involvement may aggravate the tumorigenesis of HBV-associated hepatitis without leaving the intestinal.

### *Helicobacter hepaticus* potentiated hepatocellular tumorigenesis in HBV-transgenic mice

**Fig. 2:**
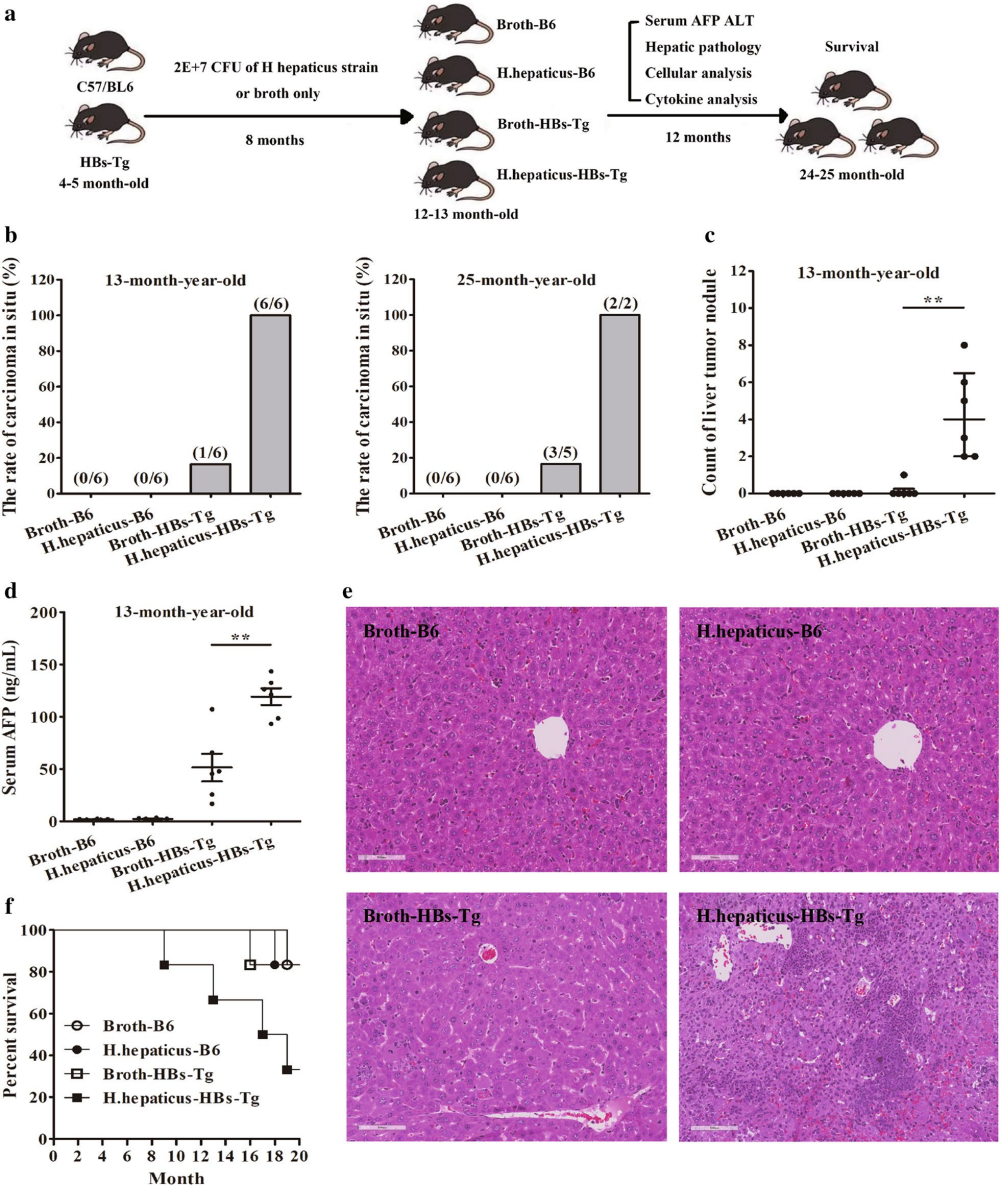
*Helicobacter hepaticus* potentiated hepatocellular tumorigenesis in HBV-transgenic mice. **a** Experimental protocol. *H. hepaticus* (2 × 10^7^ CFU) was gavaged into 4–5-month-old HBs-Tg and B6 mice every 48 h for 8 months. **b** Tumorigenesis incidence in each group (n = 6). **c** Liver tumor nodule numbers in each group (n = 6). **d** Serum AFP activity for each group (n = 6). **e** Representative H&E sections of livers in each group at 13 months old. **f** Survival of each group (n = 6). Data are expressed as median ± percentile. Mann–Whitney U and Log-rank (Mantel-Cox) test was used respectively. P < 0.05 represents statistical difference. *P < 0.05, **P < 0.01

We had observed that *H. hepaticus* enriched in intestinal were associated with not only the development of HBV-infected HCC, but advanced tumor. For defining whether *H. hepaticus* can accelerate viral hepatic tumorigenesis, we performed oral introduction of *H. hepaticus* in HBV transgenic mice. 4–5-month-old HBs-Tg mice were gavaged with *H. hepaticus* strain for 8 months (Fig. 2a). When all mice reached 13 months old, HBs-Tg mice with *H. hepaticus* administration involved in hepatic neoplasia development with a higher incidence (100%), more tumor nodules (P < 0.01) and higher serum AFP (P < 0.01), compared to wild type B6 with or without *H. hepaticus* and HBs-Tg mice (Fig. 2b–d). Hepatic histological sections exhibited, trabecular HCC with classical lobule in cirrhosis more likely appeared in HBs-Tg mice fed *H. hepaticus*, compared with the other groups (Fig. 2e) confirming the hepatocarcinoma in microcosmic. Then, neither wild type HBs-Tg mice nor wild type B6 (with or without *H. hepaticus*) at 24–25-month-old showed higher incidence for neoplasia than those HBs-Tg mice after *H. hepaticus* introduction (Fig. 2b), the survival of which was shorter than those of the other groups (P < 0.01) (Fig. 2f). Taken together, these results suggest that *H. hepaticus* can accelerate the tumorigenesis of HBV-infected hepatitis in transgenic mice.

### ILC-derived cytokines contribute to *helicobacter hepaticus*-associated HCC development in HBs-Tg mice

**Fig. 3:**
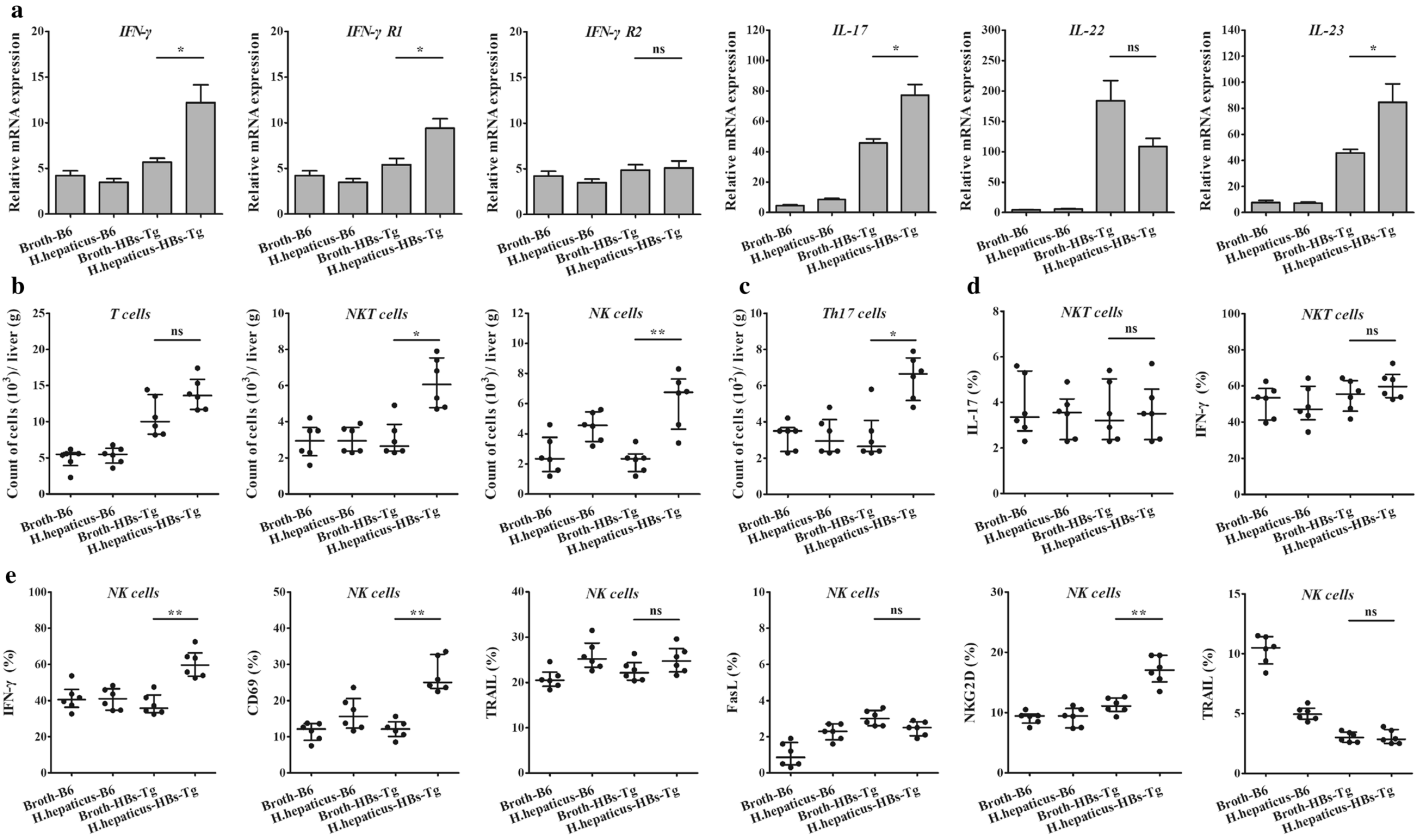
ILC-derived cytokines contribute to *helicobacter hepaticus*-associated HCC development in HBs-Tg mice. Mice (4–5 month-old) were gavaged with *H. hepaticus* strain for 8 months. At 13 months old, hepatic cytokines were tested by qRT-PCR, hepatic ILCs in mice were analyzed by flow cytometry. **a** Relative mRNA expression of IFN-γ, IFN-γR1, IFN-γR2, IL-17, IL-22 and IL-23 (n = 6 in each groups). **b** Numbers of hepatic NK cell (CD3^−^NK1.1^+^), NKT cell (CD3^+^NK1.1^+^) and T cell (CD3^+^NK1.1^−^). **c** Numbers of hepatic Th17 (CD3^+^NK1.1^−^CD4^+^IL-17^+^). Hepatic NK and NKT cells were gated to analyze the expression of phenotypical and functional molecules. **d** The expression of IFN-γ and IL-17 in intrahepatic NKT cells was analyzed by FACS. **e** The expression pattern of intrahepatic NK cells was analyzed by FACS. Data are expressed as median ± percentile. Mann–Whitney U was used. P < 0.05 represents statistical difference. *P < 0.05, **P < 0.01

It is believed that inflammatory injury of liver furthers the initiation of malignant neoplasm via inflammatory cells and their chemkines. ILCs can aggravate epithelial–mesenchymal transition during tumor progression by up-regulating inflammatory cytokines [20]. Therefore, we evaluated the cytokines expression patterns in hepatic microenvironment. We detected most cytokines which defense against viral infections (Fig. 3a and Additional file 1: Figure S1A). Significant incline in IFN-γ, IFN-γR1, IL-17 and IL-23 gene expression were found in HBs-Tg mice with *H. hepaticus* (P < 0.05) (Fig. 3a). To elucidate whether the tumorigenesis of HBV-infected hepatitis could attribute to ILCs recruited by *H. hepaticus* introduction, we characterized the infiltrating immune cells of livers in wild type B6 mice with or without *H. hepaticus*, HBs-Tg mice alone and HBs-Tg mice with *H. hepaticus* at the same time (Additional file 1: Figure S1B). NK and NKT cells, but not T cells, were enriched in the liver of HBs-Tg mice with *H. hepaticus* (T cells, P > 0.05; NKT cells, P < 0.05; NK cells, P < 0.01) (Fig. 3b). Considering the expression patterns of inflammatory fators, we more likely concerned about IFN-γ and IL-17 secreted ILCs. Then we found that, compared with the other groups, Th17 increased to a more extent in HBs-Tg mice with *H. hepaticus* introduction (P < 0.05) (Fig. 3c), while hepatic NKT cells of which were enriched without sustained increase in IL-17 and IFN-γ expression (P > 0.05) (Fig. 3d). Besides, increased expression of CD69, NKG2D and IFN-γ showed activation of cytokine production in intrahepatic NK cells in HBs-Tg mice with *H. hepaticus* (all P < 0.01), but not FasL, TRAIL and CD107a (all P > 0.05) (Fig. 3e). These results indicated that some specific ILCs activated by *H. hepaticus* may exacerbate tumorigenesis via IL-17 and IFN-γ in HBV-associated HCC.

### ILC-derived IFN-γ triggered epithelial–mesenchymal transition via STAT1 signaling in *Helicobacter hepaticus*-associated HCC

**Fig. 4:**
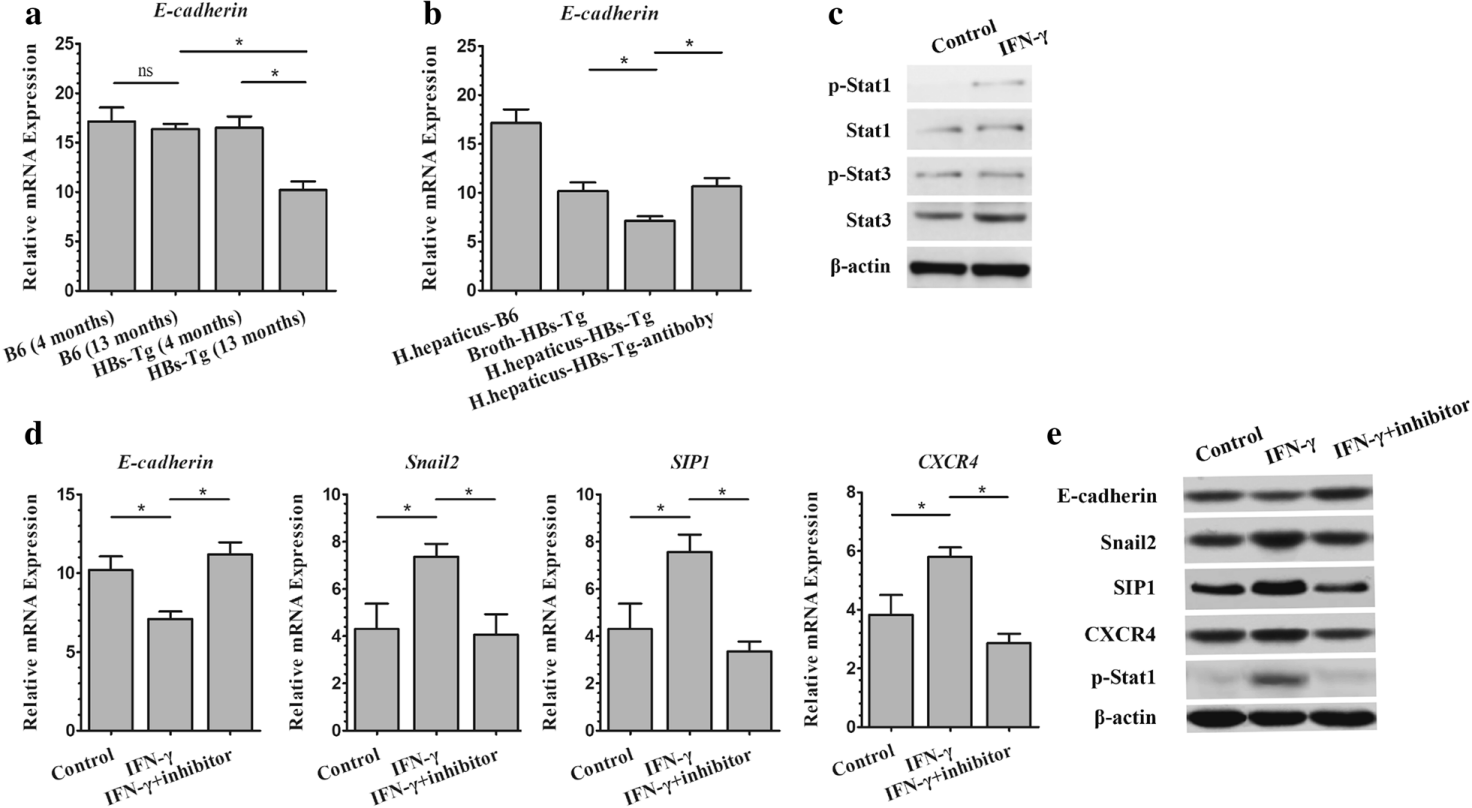
ILC-derived IFN-γ triggered epithelial–mesenchymal transition via STAT1 signaling in *Helicobacter hepaticus*-associated HCC. **a** Hepatic E-cadherin relative expression at different ages in wild type B6 and HBs-Tg. **b** Hepatic E-cadherin relative expression in *H. hepaticus* adopted HBs-Tg mice with IFN-γ antibody. **c** Hepatic signaling pathways detected by western blotting at 13 months old. **d** Detection of E-cadherin and EMT-related genes in primary hepatocytes with IFN-γ and inhibitor. Data are expressed as median ± percentile. Mann–Whitney U was used. P < 0.05 represents statistical difference. *P < 0.05, **P < 0.01

We further explored the molecular mechanism of ILC-derived IFN-γ on the tumorigenesis. The expression of E-cadherin in pure HBsTg mice gradually decreased with age (P < 0.05) (Fig. 4a), whereas, higher significantly than that in HBsTg mice with *H. hepaticus* at 13 months (P < 0.05) (Fig. 4b). IFN-γ antibody treatment significantly reversed the decrease of E-cadherin (P < 0.05) (Fig. 4b) indicated that the E-cadherin related carcinogenesis effect of *H. hepaticus* on HBs-Tg mice depended on the presence of IFN-γ. Moreover, the expression of Snail 2, SIP1 and CXCR4 was up-regulated in HBsAg-positive hepatocytes 96 h after IFN-γ stimulation in vitro (mRNA Fig. 4d, protein Fig. 4e, P < 0.05), with higher amount of p-STAT1, but not p-STAT3 (Fig. 4c). The p-STAT1 inhibitor (fludarabine) significantly reversed the change of E-cadherin, Snail2, SIP1 and CXCR4 in IFN-γ-treated HBsAg-positive hepatocytes (mRNA Fig. 4d, protein Fig. 4e, P < 0.05). The level changes of p-STAT1 protein manifested the inhibition efficiency of fludarabine in Fig. 4e. Collectively, all these data suggested ILC-derived IFN-γ repressed E-cadherin by p-STAT1 up-regulation in *Helicobacter hepaticus*-associated HCC.

## Discussion

Helicobacter species contribute to several kinds of chronic inflammation and carcinoma in mice. In spite of the presence of specific helicobacter strains were proved in human liver cancer, the pathogenic role of Helicobacter species on hepatic diseases remains undefined. *H. hepaticus* related injury and carcinogenesis in hepatocytes by activating nuclear factor-kB-regulated networks associated with innate and T helper 1-type adaptive immunity in aflatoxin B1 induced murine model of HCC [17]. However, few study investigated the involvement of *H. hepaticus* in HBV-associated HCC. In recent study, transgenic mice model with viral hepatitis mimical period were utilized to uncover the over-presented *H. hepaticus* was significantly related to the initiation of cirrhosis and neoplasm development in liver (Figs. 1 and 2). Moreover, our study indicated that *H. hepaticus* accumulation might result in the rising of the triggered anti-virus cytokines via ILCs (Fig. 3).

Hepatitis and infiltration of immune cells in *H. hepaticus*-infected cancer indicated that chronic inflammation may make primary contribution to the promotion for carcinogenesis [21]. Recruited to the liver in chronic *H. hepaticus* infected mice, plenty of neutrophils and macrophages in microenvironment accumulated oxidized nucleoside 8-hydroxydeoxyguanosine in hepatocytes by ROS secretion, exacerbating the hepatic tumorigenesis [22]. It seems that innate immunity against *H. hepaticus* is the major force attributing to this kind of inflammation [23]. Orogastric administration of *H. hepaticus* in Rag2^−/−^Apc^Min/+^ mice developed breast carcinoma, an extraintestinal tumor, in the absence of lymphocytes [24]. However, rare study has reported that NK cells can assist the malignant progress of hepatic tumors.

As we usually understood, both HCC in situ and colonic metastatic carcinoma in the liver could be restrained by phagocytosis from activated NK cells [25, 26]. In hepatitis B virus associated cancer, NK cells were guided by the cooperation between cancerous cells and virus to functionally promote HCC progression [27, 28]. This kind of role transition in molecular mechanism had been proved as increasing of inhibitory receptor on NK cells including NKG2A, Tim3 and PD-1 [29, 30]. In our study, HBV infected hepatocyte damage was initialed by reprogrammed NK cells but not NKT cells, resulting in hepatic carcinoma.

Obviously, colonization of *H. hepaticus* aggravate liver cirrhosis and neoplasia in the liver with HBV infection in previous reports [31]. Our study represents the original investigation of the intestinal microbiome in hepatic carcinogenesis without over-presentation at hepatocytes (Fig. 1). It more likely involved cytokines release reacted to *H. hepaticus* from mesenteric lymph nodes towards microenvironment in the liver. Some studies demonstrated that *H. hepaticus* associated inflammation activated innate and Th1-type adaptive immunity with inclining expression of cytokines and receptors within the intestinal lymphatic system [32].

Our study shows that *H. hepaticus* conspires with HBV to impair positive immunity of ILCs to accelerate progression of HCC. Although recent findings reveal the interaction between *H. hepaticus*, hepatic virus and liver in the evolution of HCC, further investigations are needed for novel prevention on HBV associated cancer in humans.

## Conclusion

*Helicobacter hepaticus* generate a detrimental immune microenvironment by up-regulating ILCs. ILCs-derived IFN-γ can promote the tumorigenesis of hepatitis B via E-cadherin/STAT1.

## Materials and methods

### Mice

Ten-week-old male HBV transgenic mice C57BL/6J-TgN (AlblHBV) 44 Bri (named as HBs-Tg mice) were purchased from Department of Laboratory Animal Science of Peking University. The control C57BL/6J mice are the littermates of HBs-Tg mice. All mice were housed under specific pathogen-free conditions (22 °C, 55% humidity, and 12 h day/night rhythm). Feces, colons and livers in natural HBs-Tg mice would be collected for abundance evaluation of *H. hepaticus* at 24–25 months old. Besides, they were divided into four groups characterised by the presence or absence of the HBV transgene and *H. hepaticus* infection (shown as Fig. 1). Beginning at 4–5-month-old, mice were gavaged with 2 × 10^7^ colony-forming units (CFU) of *H. hepaticus* strain or broth only every 48 h for 8 months as previously described [33]. Mice were euthanatised at 12–13 or 24–25-month-old by CO_2_ inhalation. Blood and livers were harvested at 12–13-month-old, submitted for histopathology, serum test, and quantitative real-time PCR (qRT-PCR). At 24–25-month-old, survival rate would be calculated.

### Bacterial strains

*Helicobacter hepaticus* (type strain ATCC 51448) was cultured according to previously described [33]. Briefly, they grew under microaerobic conditions (37 °C) using trypticase soy blood agar (BD Biosciences, USA) at first, followed by inoculation in 5% fetal bovine serum-contained brucella broth on a rotary shaking incubator (Thermo Fisher Scientific, USA) for 48 h. Then, the cultured broth was centrifuged at 10,000 rpm (4 °C) for 20 min. After harvested in exponential phase using OD600 nm test, the pellet was resuspended in brucella broth containing 30% glycerol, to approximately 10^8^ organisms/ml as confirmed by spectrophotometry (MEGATOO, Beijing, China). Oral gavage of 0.2 ml fresh culture was administrated into examed mice for three doses every 2 days, while medium alone was introduced into controls synchronously. Subculture of the inoculum and the medium on blood agar was used for maintaining the purity of the strain and the sterility of the medium.

### Serum biochemical assays

Serum alanine aminotransferase (ALT), alpha-fetoprotein (AFP) was determined using ELISA kits (R&D systems, Minneapolis, MN), according to the manufacturer’s instructions.

### Preparation of fecal sample and colon tissues for PCR

Based on the manufacturer’s instructions, DNA in feces was extracted using the QIAamp DNA stool Mini Kit (Qiagen, USA) with a spin column, followed by an elution in the Tris–EDTA buffer (pH 8). DNA of intestinal microbiome in situ was isolated from frozen colorectal tissues after mechanical homogenization (Tissue Lyser, Qiagen, USA), using the EZ1 DNA Tissue kit and the EZ1 BioRobot (Qiagen, USA). The parameter of the extracted DNA was measured by the NanoDrop 2000 spectrophotometer (Thermo Scientific, USA). All DNA samples were stored at − 80 °C.

### Quantitative PCR

All reactions were assayed in 20 μL reaction volume containing 1× final concentration TaqMan Universal Master Mix (Applied Biosystems, USA) in a 96-well optical PCR plate. Each reaction contained 5 ng of extracted faecal DNA and 5 μM of primers. Amplification and detection of DNA was performed with the Roche Lightcycler 480 Quantitative Analysis System (Applied Biosystems). the following reaction conditions would be applied: 2 min at 50 °C, 10 min at 95 °C, and 40 cycles of 15 s at 95 °C and 1 min at 60 °C. The primers for detecting *H. hepaticus* and total bacteria were used as previously described [34]. The primers’ sequences were as follows:

*H. hepaticus* forward primer, 5′-GCAUUUGAAACUGUUACUCUG-3′;

*Helicobacter hepaticus* reverse primer, 5′-GGGGAGCUUGAAAACAG-3′;

Total bacterial DNA forward primer, 5′-GCAGGCCTAACACATGCAAGTC-3′;

Total bacterial DNA reverse primer, 5′-CTGCTGCCTCCCGTAGGAGT-3′.

Every sample was assayed in triplicate in same batch, and the mean of these cycle threshold (Ct) values was calculated for subsequent study. The relative abundance of *H. hepaticus* normalised to the total bacteria of each sample was calculated using the 2^−ΔCt^ method (where ΔCt = the average Ct value of *H. hepaticus* in each sample—the average Ct value of total bacteria).

All primers of cytokines in livers were synthesized by Invitrogen (Thermo Fisher Scientific, USA), as shown in Additional file 1: Table S1. Results were analyzed using the ΔΔCt method and β-actin as the reference.

### Primary mouse hepatocyte isolation and culture

Details are shown in supplementary materials and methods. The isolation and culture of primary mouse hepatocytes were performed as described [35]. IFN-γ (PeproTech, USA) treatment was using 2 ng/ml. STAT1 activity inhibition was done by incubation with 20 μM Fludarabine (Selleckchem, USA) for 24 h, followed by IFN-γ treatment for another 24 h.

### Western blotting

Details are shown in supplementary materials and methods. Liver tissues were lysed as described [35]. After SDS-PAGE, proteins were transferred onto PVDF membranes (Millipore Corporation, USA), and incubated with primary Abs over-night at 4 °C. Membranes were washed with 0.1% (vol/vol) Tween 20 in TBS (pH 7.6) and incubated with a 1:2, 500 dilution of horseradish peroxidase-conjugated secondary Abs for 60 min at room temperature. Protein bands were visualized by ECL reaction (Pierce Biotechnology, Rockford, IL).

### Isolation of mononuclear cells in hepatic tumor

Hepatic mononuclear cells were prepared as previously described [35]. Briefly, liver of mice was removed and washed with Ca and Mg free Dulbecco’s Phosphate Buffered Saline (DPBS). After pressing through a 200-gauge stainless steel mesh, the cell mixture was resuspended in 40% Percoll solution (General Electric Company, USA), followed by overlaying gently onto 70% Percoll solution. Then, this cell mixture was centrifuged at 1260×*g* for 30 min at room temperature. The interface cells between the percoll solutions were aspirated and washed twice with PBS medium. Single cell suspensions were resuspended in cell staining solution (PBS with 2% FCS) for flow cytometry.

### Multicolor flow cytometry analysis

After Fc receptors blocking (BD Biosciences, USA), an appropriate concentration of the fluorescence-labeled antibody was used for the staining of surface antigens at 4 °C for 30 min in dark place. Fluorochrome-conjugated monoclonal antibodies of cellular markers: PercpCy5.5-anti-CD3, FITC-anti-IL-17A, PE-anti-CD4, PE-Cy7-anti-NK1.1 (BD Bioscience, USA). FITC-anti-CD69, FITC-anti-IFN-γ; PE-anti-FasL, PE-anti-TRAIL, PE-anti-CD107a, and APC-anti-NKG2D (eBioscience, San Diego, CA). The intracellular cytokine staining, including INF-γ and IL-17A, was using Mouse Intracellular Cytokine Staining Starter Kit (BD Biosciences, USA), according to the kit’s instructions. Samples were measured by a BD Accuri C6 plus flow cytometer (BD Biosciences, USA), and the data were managed using BD Accuri C6 plus analysis (BD Biosciences, USA).

### Statistical analysis

All statistics were performed using GraphPad Prism 5.0 software (La Jolla, CA). Results of inflammatory genes, tumor number, ALT activity, AFP activity, cellular analysis, survival analysis and richness of bacteria were analyzed using Mann–Whitney U test, Log-rank (Mantel–Cox) test as appropriate. Data are expressed as the medians with interquartile range. P < 0.05 was considered significant in comparison of medians.

## Additional file

**Additional file 1: Figure S1.** Innate lymphoid cells contribute to *helicobacter hepaticus*-associated HCC development in HBs-Tg mice. **Table S1.** The primers for each gene detected by real-time PCR.

## Data Availability

The datasets analyzed during the current study are available from the corresponding author on reasonable request.
