# Supplementary material for: Cytokines derived from innate lymphoid cells assist Helicobacter hepaticus to aggravate hepatocellular tumorigenesis in viral transgenic mice
Source: Gut Pathog. 2019 May 15;11:23. doi: 10.1186/s13099-019-0302-0 (PMC6521485; doi:10.1186/s13099-019-0302-0)
Supplement: Supplementary file 1 — Additional file 1: Figure S1. Innate lymphoid cells contribute to helicobacter hepaticus-associated HCC development in HBs-Tg mice. Table S1. The primers for each gene detected by real-time PCR. [file 13099_2019_302_MOESM1_ESM.docx]

**Additional file 1**


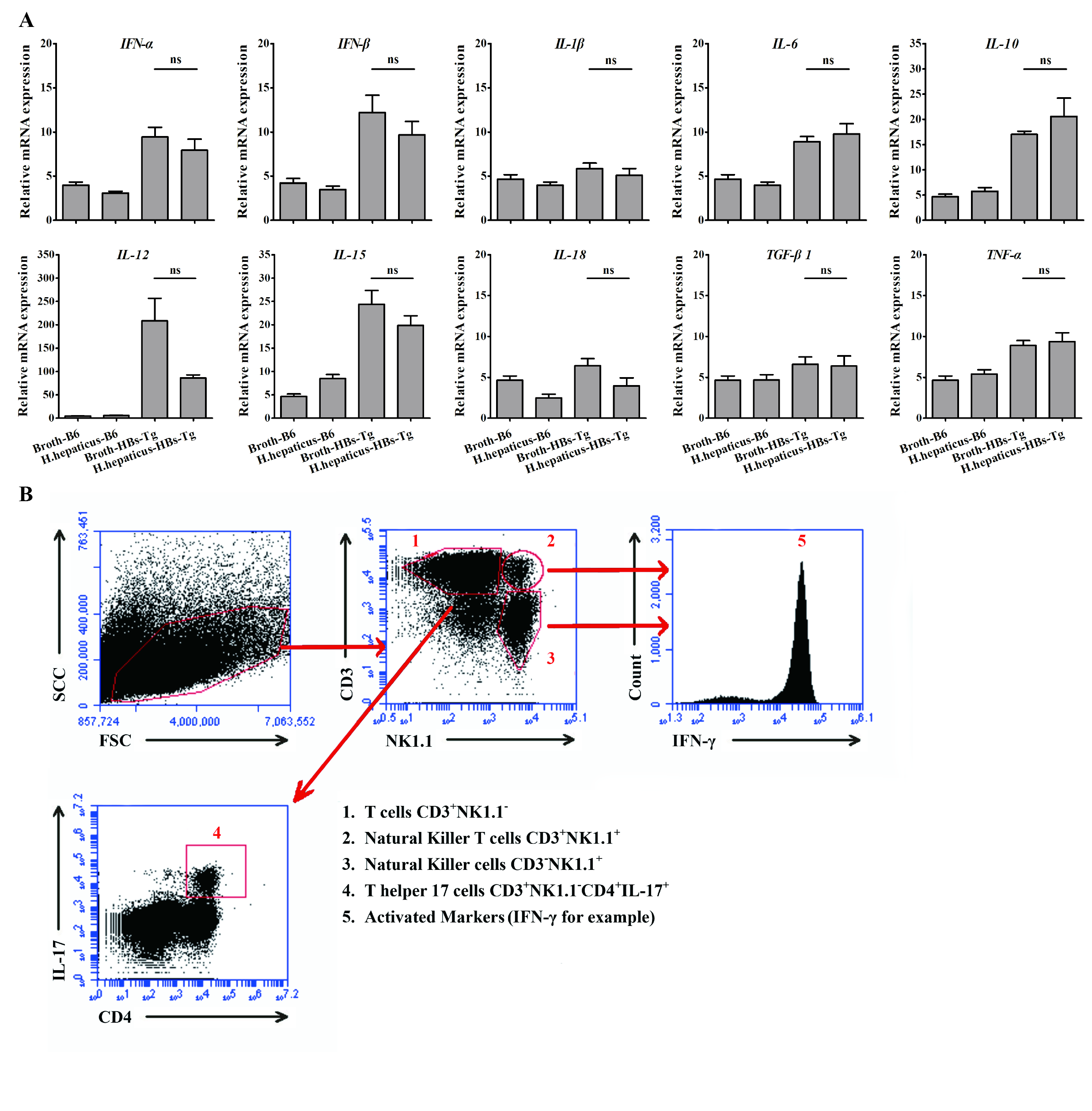


**Figure S1. Innate lymphoid cells contribute to *helicobacter hepaticus*-associated HCC development in HBs-Tg mice**

**(A)** Relative mRNA expression of IFN-α/β, IL-1β, IL-6, IL-10, IL-12, IL-15, IL-18, TGF-β1, TNF-α (n=6 in each groups). **(B)** A schematic of the gating strategy for subset identification of innate lymphoid cells. Data are expressed as median ± percentile. Mann-Whitney U was used. P <0.05 represents statistical difference. * P <0.05 ** P <0.01

| *Gene* | *Forward primer (5’-3’)* | *Reverse primer (5’-3’)* |
| --- | --- | --- |
| *IFN-α* | TCTGATGCAGCAGGTGGG | AGGGCTCTCCAGAYTTCTGCTCTG |
| *IFN-β* | GTCCTCAACTGCTCTCCACT | CCTGCAACCACCACTCATTC |
| *IFN-γ* | ACTGGCAAAAGGA TGGTGAC | TGAGCTCATTGAATGCTTGG |
| *IFN-rRI* | GGTTGCTCCTCTTACCGTCT | AGGGGCTCTTCACAGATCAC |
| *IFN-rRII* | CTCCCCTCCCTTTGATGTGT | TGCTGTTGTTTCGTGACAGG |
| *IL-1β* | GACCTTCCAGGATGAGGACA | AGGCCACAGGTATTTTGTCG |
| *IL-6* | AACGATGATGCACTTGCAGA | GGAAATTGGGGTAGGAAGGA |
| *IL-10* | GGTGAGAAGCTGAAGACCCT | TGTCTAGGTCCTGGAGTCCA |
| *IL-12* | GATGACATGGTGAAGACGGC | AGGCACAGGGTCATCATCAA |
| *IL-15* | GAGGCTGGCATTCATGTCTT | GCAA TTCCAGGAGAAAGCAG |
| *IL-17a* | TCTCTGATGCTGTTGCTGCT | AGCA TCTTCTCGACCCTGAA |
| *IL-18* | GACAAAGAAAGCCGCCTCAA | GTGAAGTCGGCCAAAGTTGT |
| *IL-22* | CCGAGGAGTCAGTGCTAAGG | AGCTTCTTCTCGCTCAGACG |
| *IL-23* | ATGCTGGATTGCAGAGCAGTA | ACGGGGCACATTATTTTTAGTCT |
| *TGF-β1* | ATTCAGCGCTCACTGCTCTT | TCTCTGTGGAGCTGAAGCAA |
| *TNF-α* | CCACATCTCCCTCCAGAAAA | AGGGTCTGGGCCATAGAACT |
| *β-actin* | TGACGTTGACATCCGTAAAGACC | CTCAGGAGGAGCAATGATCTTGA |
| *Snail2* | TTCTACGTTCTCTGGGCTGG | GCAGTGAGGGCAAGAGAAAG |
| *SIP1* | ACTGGAGGAAAGAGATGGCC | CTCCTTCAGCGATGTCAAGC |
| *CXCR4* | GAAGTGGGTTCTGGAGACT | TGTGA TGACAAAGAGGAGGT |
| *Ecadherin* | GAGGTCTACACCTTCCTGGTG | TCTGTAGACACTTTGAATCGG |

**Table S1. The primers for each gene detected by real-time PCR**
